# A community-based study of the relationship between calcaneal bone mineral density and systemic parameters of blood glucose and lipids

**DOI:** 10.1097/MD.0000000000016096

**Published:** 2019-07-05

**Authors:** Li-juan Gu, Xiao-yang Lai, You-ping Wang, Jian-min Zhang, Jian-ping Liu

**Affiliations:** a Department of Endocrinology, Fengcheng People's Hospital of Jiangxi Province; b Department of Endocrinology, Second Affiliated Hospital of Nanchang University, China.

**Keywords:** bone mineral density (BMD), fasting blood glucose (FBG), high-density lipoprotein cholesterol (HDL-C), low-density lipoprotein cholesterol (LDL-C), Osteoporosis (OP), total cholesterol (TC), triglyceride (TG)

## Abstract

Osteoporosis (OP) is a disease characterized by decreased bone mineral density (BMD) and an increased risk of osteoporotic fractures. Nutritional factors (including glucose and fats lipids), have been implicated in OP.

We hypothesized that the levels of blood glucose and lipids could be biomarkers for predicting the risk of OP. To test this hypothesis, we evaluated the potential relationship between BMD and levels of blood glucose and lipids via a community-based study in China.

This was a community-based cross-section analysis, and a total of 8584 cases were investigated. The BMD of the left calcaneus was measured using an ultrasonic bone densitometer. The levels of blood glucose (fasting blood glucose [FBG], 2-h blood glucose [2hBG], and glycosylated hemoglobin [HbAlc]), and lipids (triglyceride [TG], total cholesterol [TC], low-density lipoprotein cholesterol [LDL-C], and high-density lipoprotein cholesterol [HDL-C]) were measured and analyzed.

In our study population, the levels of FBG, 2hBG, HbAlc, TC, LDL-C and HDL-C were higher in the OP group than in the low bone density and the normal bone density groups, while the levels of HbAlc, TC, and LDL-C in the low bone density group were higher than those in the normal bone density group. In males, the level of blood LDL-C in the low bone density group was higher than that in the normal bone density group. In postmenopausal subjects, the levels of FBG, 2hBG and HbA1C were higher than those in the normal bone density groups, and the level of HbA1C in the low bone density group was higher than that in the normal bone density group. Pearson linear trend analysis demonstrated that BMD was positively associated with TC and LDL-C in males and negatively associated with FBG, 2hBG and HbA1C in postmenopausal females. Moreover, logistic analysis showed that BMD was correlated with TC in premenopausal females and HbA1C in postmenopausal females.

OP is generally associated with abnormal levels of blood glucose and/or lipids; nevertheless, the relationship between OP and abnormal levels of blood glucose and/or lipids is complicate and different subpopulations may have different susceptibilities. Therefore, further detailed studies are warranted.

## Abstract 2

Supplemental Digital Content is available in the text

## 1 Introduction

Osteoporosis (OP) is a disease characterized by decreased bone mineral density (BMD) and an increased risk of osteoporotic fractures. In fact, OP is the most common cause of bone breaks, and osteoporotic fractures are the major causes of disability and death in elderly patients. Mechanistically, numerous potential risk factors, such as aging, nutritional factors, and hormones (especially sex hormones), have been implicated in OP. Among many nutritional factors, sugars (glucose) and fats (lipids) are thought to play crucial functions in bone metabolism and maintaining bone health; unsurprisingly, disorders in glucose and lipid metabolism, such as hyperglycemia, hyperlipidemia, diabetes, atherosclerosis, and hypercholesterolemia, have all been associated with OP to different degrees. For example, a previous study suggested that an increased low-density lipoprotein cholesterol (LDL-C) level is associated with non-vertebral fractures in postmenopausal women.^[1]^ Consistently, most researchers have reported that consumption of a high-fat diet is associated with low BMD, decreased bone strength, adverse microstructure changes in the cancellous bone compartment, an altered bone marrow environment, and low grade inflammation in the elderly.^[2]^ However, another report indicated that hyperglycemia is associated with increased BMD and a decreased trabecular bone score in elderly men^[3]^; nevertheless, the exact relationship between BMD and the levels of blood glucose and lipids is still largely unknown. Furthermore, few community-based, large scale studies, which have the potential to indicate a potential association the dynamic relationship between BMD and blood glucose and lipids in different subpopulations, have been reported.

For the above-mentioned reasons, we hypothesized that the levels of blood glucose and lipids could be biomarkers for predicting the risk of OP. To test this hypothesis, we performed a community-based study of the relationship between the calcaneal BMD and blood glucose and lipid levels in a Chinese population. We use calcaneal quantitative ultrasound, instead of standard BMD quantification via dual-energy X-ray absorptiometry at the femoral neck (FN) and/or lumbar spine (LS), to assess BMD, because this approach is easy to scale up, streamline and relatively low cost. Accordingly, quantitative ultrasound has emerged as a convenient and popular screening tool for osteoporosis. Supporting this idea, a previous report demonstrated that calcaneal quantitative ultrasound measurement is a valid and independent predictor of fracture risk similar to, but slightly different from, standard BMD measurement, as defined by the World Health Organization (WHO) criteria.^[2,4]^ Another clinical study also found that quantitative ultrasound indices not only were associated with BMD but also could discriminate subjects with and without a fracture history and predict the risk for future fracture.

The results of the present study support the idea that BMD is closely associated with abnormalities in glucose and lipid metabolism in general, although a more comprehensive understanding is needed to provide a solid basis for the development of a novel paradigm to improve bone health and treat OP via modifying glucose and lipid components.

## 2 Subjects and methods

### 2.1 Subjects

This study selected permanent residents of 15 communities in Nanchang, Jiangxi Province who were at least 40 years old as the research subjects (residents who were <40 years old were excluded because OP or osteopenia is only consistently found in adults over 40 years of age).

Participants were first categorized according to sex. Then women who had ceased menstruation for at least 12 months were grouped in the postmenopausal group, and the other women were included in the premenopausal group. Residents were excluded if they met the following criteria:

1. pregnant or lactating;
2. used drugs that might affect BMD, such as vitamin D, bisphosphonates, estrogen, male hormones, glucocorticoids, immunosuppressive agents, and thyroid hormones;
3. diabetic;
4. suffered from diseases that may affect BMD, such as parathyroid disease, thyroid disease, gout, rheumatoid immune disease, cancer, severe cardiovascular (including confirmed hypertension patients who receive daily drug treatment. In contrast, patients with mild borderline hypertension who receive no regular drug treatment were included) and cerebrovascular diseases as well as severe liver and renal insufficiency diseases; and
5. had only incomplete data, including the 103 cases of drop out and 212 cases with missing data.

Overall, a total of 8584 cases with complete data were investigated. The enrolled subjects showed good compliance with the study, and all signed an informed consent form. The experimental protocol was established according to the ethical guidelines of the *Declaration of Helsinki* and approved by the Human Ethics Committee of Second Affiliated Hospital of Nanchang University, China.

### 2.2 Research methods

Trained doctors conducted surveys using standardized questionnaires to collect data on lifestyle (cigarette smoking, wine drinking, milk drinking, and sports activities), disease history and drug treatment history. They also measured the basic parameters of the human body, including body height and weight, and calculated the body mass index (BMI).

### 2.3 Determining the levels of sugars and lipids in blood

All subjects received an oral glucose tolerance test (OGTT) after fasting for 10 hours. Blood samples were collected from the elbow vein, and FBG, 2-hr blood glucose (2hBG), total cholesterol (TC), triglyceride (TG), LDL-C, and high-density lipoprotein cholesterol (HDL-C) levels were measured using an automatic biochemical analyzer (Siemens ADVIA Centaur XP automatic biochemical analyzer, Germany). HbAlc was measured via high-pressure liquid chromatography (by Dimension RL Max special protein analyzer).

### 2.4 BMD measurement

The Sahara ultrasound BMD analyzer (HOLOGIC, USA), was used to quantitatively measure the BMD of the left calcaneus of each subject. The analyzer was used to establish a database for the healthy Chinese population, and the data were directly converted into T values. The ultrasound BMD analyzer was operated by dedicated trained persons, and the machine was tested and corrected with a special program before the daily testing. In each individual case, the gender of the participant was entered, and then the BQI (bone index) and automatically converted to a T value based on the measured data.

The following diagnostic criteria were applied: a BMD reduction within one standard deviation of the peak bone mass of normal adults of the same sex and race (T>−1) was considered to be normal; a BMD reduction between 1 and 2.5 standard deviations (i.e., −2.5 < T≤−1) was considered to be osteopenia; and a BMD reduction equal to and greater than 2.5 standard deviations was considered to be OP.

### 2.5 Statistical methods

SPSS 19.0 software was used for analysis. Qualitative data are expressed as numbers of positive cases. Quantitative data are expressed as means ± standard deviations. The *t* tests were used to test the statistical significance of a difference between two groups, and count data were tested using chi-square test. One-way analysis of variance (ANOVA) was used for intergroup comparisons. Logistic analysis was used to analyze the correlations between the indexes. *P* < .05 indicated that the difference was statistically significant.

## 3 Results

### 3.1 General information

Among the 8584 tested subjects, 2830 were male and 5754 were female. Among the females, 2209 were premenopausal and 3545 were postmenopausal (premenopausal: postmenopausal = 1:1.6). According to the bone density T value, the subjects were divided into 3 groups: a normal BMD group (T>−1.0; 4274 cases), an osteopenia group (−2.5<T≤−1; 3551 cases), and an osteoporosis group (T≤−2.5; 759 cases). The sex ratio was not significantly different among the different groups, but the average age and body mass index (BMI) differed among many of the groups, creating a possible confounding factor that should be taken into consideration in the final interpretation of the results (Table 1).

**Table 1:**
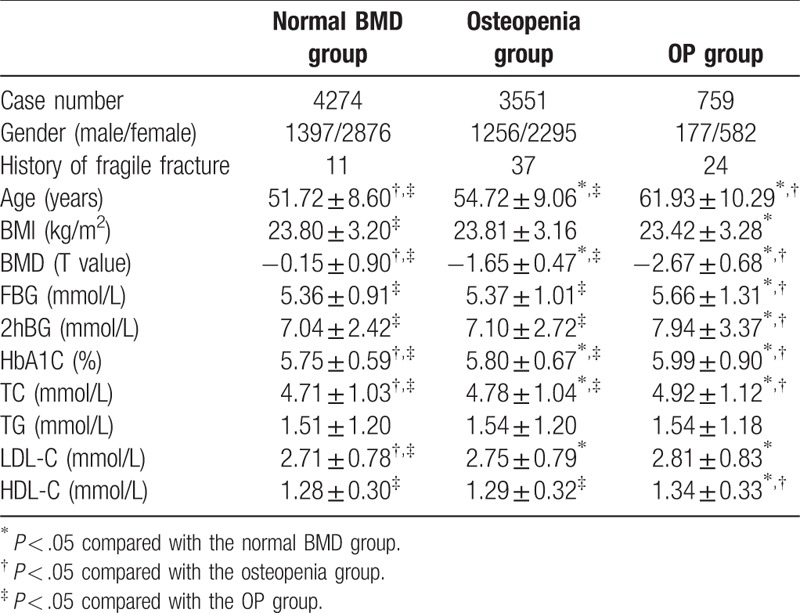
Comparison of clinical parameters among the 3 BMD groups 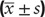.

### 3.2 Association of BMD values with blood glucose and lipid levels within the general population

The analysis based on the entire population showed that, among different BMD groups, the levels of blood glucose (FBG, 2hBG and HbAlc) and lipids (TC, LDL-C and HDL-C) were also different; that is, in the OP group the levels of blood glucose and lipids were generally higher than those in the normal BMD and osteopenia groups. The only exception was for TG, which did not significantly vary with changes in BMD values. These data suggest that, generally, there is an intimate relationship between BMDs and the indicators of blood glucose and lipids (Table 1).

### 3.3 Differences in the male and female populations

To further dissect the interrelationship between BMD and the levels of blood glucose and lipids and to clarify the subpopulation-specific phenotype, we next focused on the male population only. We found that, among different BMD groups, the levels of blood glucose and lipids were not consistently changed, except that the HDL-C level in the OP group was significantly higher than those in the normal BMD and osteopenia groups (*P* < .05; Table 2). In great contrast, within the female population, there was a close relationship between BMD measurements and the indicators of blood glucose and lipids; that is, among different BMD groups, the levels of blood glucose and lipids were also consistently altered (*P* < .05; Table 2).

**Table 2:**
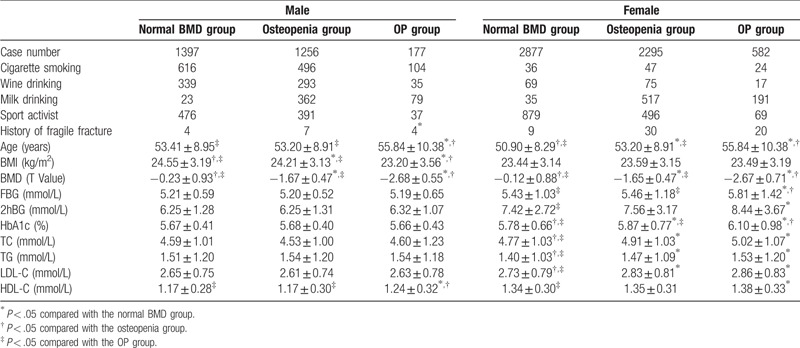
Comparison of the clinical parameters among the three BMD groups for the male and female populations, separately 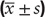.

### 3.4 Differences in the premenopausal and postmenopausal populations

More interestingly, among premenopausal women, there was no obvious interrelationship between BMD and the indicators of glucose and lipid levels (*P* > .05), whereas for the postmenopausal population, among different BMD groups, the blood glucose levels (FBG, 2hBG, and HbA1C) were consistently changed (*P* < .05), but the lipids indicators were not (Table 3).

**Table 3:**
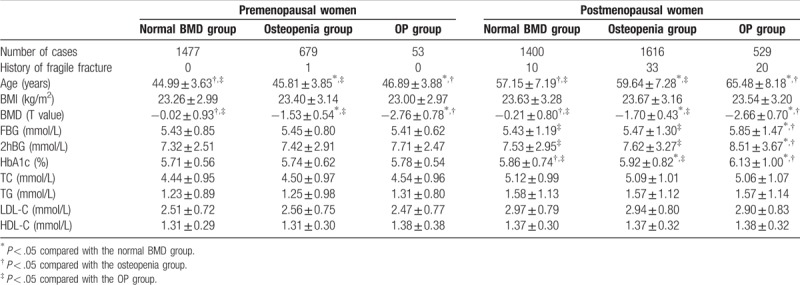
Comparison of the clinical parameters among the three BMD groups for premenopausal and postmenopausal females, separately 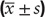.

### 3.5 Logistic analysis to identify potential risk factor(s) for low BMD

T values were considered as the dependent variable, while FBG, 2hBG, HbA1C, TC, TG, LDL-C, and HDL-C were considered as the independent variables. After correction for the potential involving factors, such as age, BMI, cigarette smoking, wine drinking, coffee drinking, milk drinking, and regular exercises, logistic analysis (backward) showed that BMD was correlated with TC in premenopausal females and HbA1C in postmenopausal females (*P* < .05; Tables 4–6, suppl. Table 1).

**Table 4:**
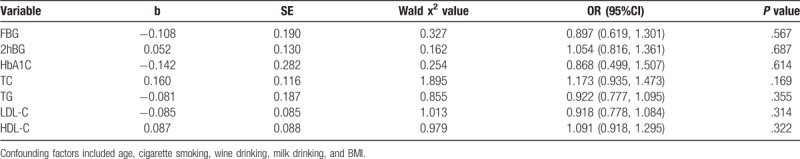
Multivariate logistic regression analysis of associations between glycemic parameters and lipid profiles with BMD in male participants, after correction for confounding factors.

**Table 5:**
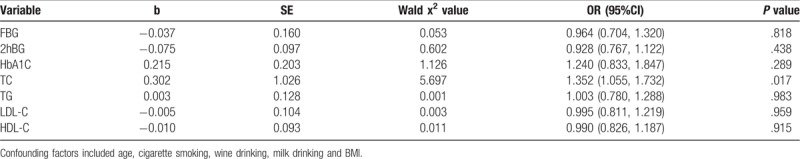
Multivariate logistic regression analysis of associations between glycemic parameters and lipid profiles with BMD in premenopausal women after correction for confounding factors.

**Table 6:**
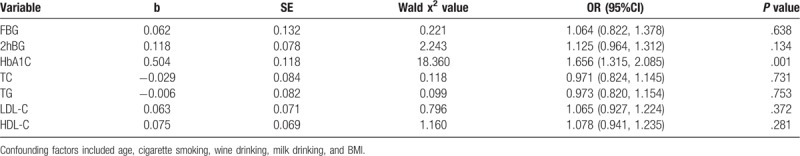
Multivariate logistic regression analysis of the associations of glycemic parameters and lipid profiles with BMD in postmenopausal women after correction for confounding factors.

### 3.6 Receiver operating characteristic (ROC) analysis

**Figure 1:**
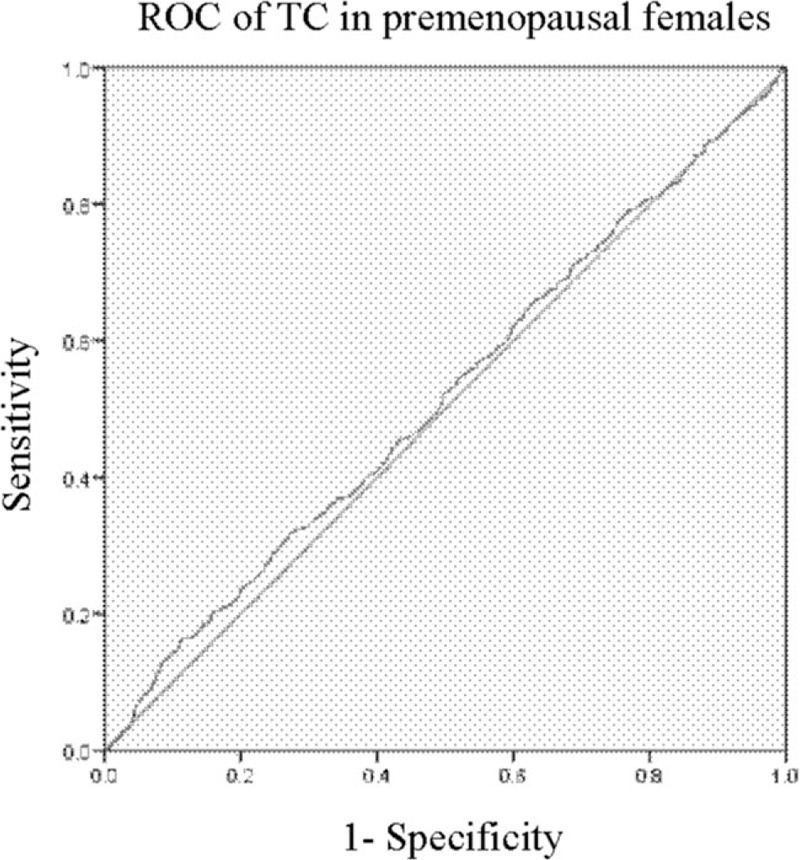
ROC for TC in premenopausal females. The AUC was 0.518 with poor sensitivity and specificity, *P* > .05. TC = total cholesterol.

**Figure 2:**
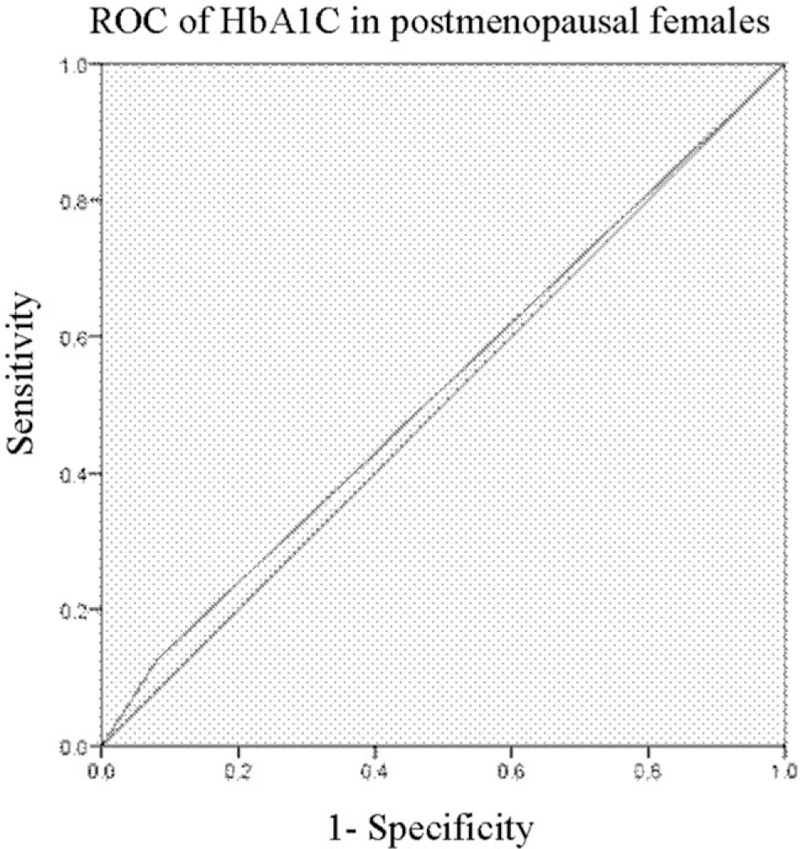
ROC for TC in premenopausal females. The AUC was 0.523 with good sensitivity and specificity, *P* < .05. TC = total cholesterol.

Logistic analysis (backward) showed that BMD was correlated with TC in premenopausal females and HbA1C in postmenopausal ones. To identify their sensitivities and specificities, we performed a ROC curve analysis. The area under the curve (AUC) for TC in premenopausal females was 0.518 with poor sensitivity and specificity, *P* > .05 (Fig. 1). In contrast, the AUC for HbA1C in postmenopausal females was 0.523 with sensitivity and specificity, *P* < .05 (Fig. 2).

## 4 Discussion

This study intended to identify potential glucose and lipid levels as risk factors for low BMD, and the data from our study support the idea that disorders of glucose and lipids might be closely associated with OP or osteopenia, suggesting high levels of glucose and lipids might be risk factors for OP. However, it is worth noting that this cross-sectional study did not intend to establish a causal or direct relationship between BMD and indicators of glucose and lipid levels; rather this study intended to identify potential glucose and lipid levels as risk factors for low BMD. This community-based study selected healthy adults over 40 years of age as the research subjects, since OP or osteopenia has been only consistently found in adults over 40 years of age. Our analysis identified some general trends:

1) not all indicators are equally useful, and the same indicator might be associated with BMD in some subpopulations but not in others. For example, the elevated FBG was a potential risk factor for decreased BMD in postmenopausal women but was not a useful indicator of BMD for men or premenopausal women.
2) It is possible that different methods might identify different risk factors. For example, logistic analyses, compared to ANOVA or *t* test, identified different potential risk factors, although sometimes these factors might overlap with each other. Therefore, this study also raised some questions.

Furthermore, even though the statistically significant changes were associated with a certain BMD, it is still unclear whether the changes are really biological meaningful; especially when the heterogeneity of the population (within a certain BMD group) are taken into consideration.

Nevertheless, one consistent finding was that indicators of glucose level could be risk factors for BMD reduction, especially in postmenopausal women. Lohmander et al^[5]^ reported that hyperglycemia is often associated with osteopenia and OP, and Anaforoglu et al^[6]^ also found that the incidence of brittle fracture in type 2 diabetes patients with poor blood glucose control is significantly higher than that in non-diabetic patients. The underlying mechanisms could be:

1. decreased insulin secretion and/or decreased insulin sensitivity, resulting in elevated blood glucose levels.^[7,8]^ The high blood sugar levels lead to the accumulation of advanced glycation end products on collagen, undermining the balance between bone resorption and bone formation.^[9]^
2. Hyperglycemia causes diuresis and increases phosphorus calcium output, which, in turn, stimulates parathyroid secretion of parathyroid hormone and increases the osteolytic effect, resulting in a reduction in BMD.^[10]^
3. The hyperglycemic state causes tissue hypoxia and microvascular lesions, destroying the bone microstructure and affecting bone reconstruction.^[11]^
4. There were significant differences in blood glucose levels among the postmenopausal female groups with different BMDs, which may be related to the decrease in estrogen levels in postmenopausal women, which affects insulin sensitivity and insulin resistance and results in increased blood glucose levels.^[12]^

To put our study in a proper perspective, we briefly reviewed the relevant literature. For example, previous studies have led to inconsistent conclusions regarding the correlation between BMD and lipid metabolism. Sivas et al^[13]^ and Tarakida et al^[14]^ found that elevated cholesterol levels in postmenopausal women can accelerate the loss of bone mass, resulting in decreased BMD. Trimpou et al^[15]^ observed a necrotic femoral head under electron microscopy and found significantly increased numbers and sizes of fat cells, suggesting that hypercholesterolemia is an independent risk factor for osteoporotic fractures. However, Sivas et al^[13]^ suggested that TG was not associated with BMD in postmenopausal women. Ersoy et al^[16]^ and Makovey et at^[17]^ found that TC and LDL-C levels in postmenopausal women with OP were lower than those in postmenopausal women with a normal BMD by different studies. Pliatsika et al^[18]^ found no correlation between cholesterol, TG, and LDL-C levels and BMD in postmenopausal women. Regarding the relationship between HDL-C and BMD, different studies also have yielded different results.^[19]^ Hyder et al^[20]^ suggested that HDL-C and the BMD of females were not correlated, but Jeon et al^[21]^ found that HDL-C levels in OP patients were positively correlated with lumbar BMD. Overall, the results from multiple clinical studies are inconsistent, which may be due to differences in the races and genders of the subject populations and the methods and sites of BMD measurement.

Our current study has some limitations. For example, although the sample size of this study was large, the proportion of people with abnormal glucose and lipid metabolism was generally small. Also, potential confounding factors included uneven average age and BMI baseline in the different BMD groups, which may interfere with the final interpretation, and possibly explain why the results of this study are not completely consistent with previous reports.

## 5 Conclusion

The levels of blood glucose and lipids in the OP group were generally higher than those in the normal BMD and osteopenia groups. Further analysis demonstrated that the BMD of males was correlated positively with LDL-C, while the BMD of female was negatively correlated with FBG and HbA1c, although postmenopausal and premenopausal females showed different trends. Our study suggests that abnormal glucose and lipid levels might be among the risk factors for OP; nevertheless, different subpopulations may have differing susceptibilities to abnormalities in glucose and lipid levels; therefore, further detailed studies are warranted.

## Acknowledgments

We thank all the participants for their willingness to participate in this study.

## Author contributions

**Conceptualization:** Li-juan Gu, Xiao-yang Lai, Jianping Liu.

**Formal analysis:** Li-juan Gu.

**Funding acquisition:** Li-juan Gu.

**Investigation:** Li-juan Gu.

**Methodology:** Li-juan Gu, You-ping Wang, Jian-min Zhang.

**Project administration:** You-ping Wang, Jianping Liu.

**Resources:** Jianping Liu.

**Software:** Li-juan Gu, Xiao-yang Lai, You-ping Wang, Jianping Liu.

**Supervision:** You-ping Wang, Jian-min Zhang.

**Validation:** Xiao-yang Lai, You-ping Wang, Jianping Liu.

**Visualization:** Xiao-yang Lai, Jianping Liu.

**Writing – original draft:** Li-juan Gu, You-ping Wang, Jianping Liu.

**Writing – review & editing:** Jian-min Zhang.

## Supplementary Material

Supplemental Digital Content
