# Supplementary material for: A community-based study of the relationship between calcaneal bone mineral density and systemic parameters of blood glucose and lipids
Source: Medicine (Baltimore). 2019 Jul 5;98(27):e16096. doi: 10.1097/MD.0000000000016096 (PMC6635260; doi:10.1097/MD.0000000000016096)
Supplement: Supplemental Digital Content [file medi-98-e16096-s001.doc]

**Supplementary Table 1** Assignments of variables in the analysis of risk factors for BMD through a logistic regression model

| ***Variable*** | ***Assignments*** |  |
| --- | --- | --- |
| ***Age*** | actual value |  |
| ***Wine drinking*** | yes=1; n=0 |  |
| ***Cigarette smoking*** | yes=1; n=0 |  |
| ***Sports activity*** | yes=1; n=0 |  |
| ***BMI (kg/m^2^)*** | <24.0 kg/m^2^=0; ≥24.0 kg/m^2^=1 |  |
| ***FBG (mmol/L)*** | <6.1 mmol/L=1; ≥6.1 mmol/L=0 |  |
| ***2hBG (mmol/L)*** | <7.8 mmol/L=0; ≥7.8 mmol/L=1 |  |
| ***HbA1C (%)*** | <6.5%=0; ≥6.5%=1 |  |
| ***TC (mmol/L)*** | <5.47 mmol/L=0; ≥5.47 mmol/L=1 |  |
| ***TG (mmol/L)*** | <1.7 mmol/L=0; ≥1.7 mmol/L=1 |  |
| ***LDL-C (mmol/L)*** | LDL-C <2.6 mmol/L=0; LDL-C ≥2.6 mmol/L=1 |  |
| ***HDL-C (mmol/L)*** | ≥1.03 mmol/L(M) or 1.29 mmol/L (F)=0; <1.03 mmol/L (M) or 1.29 mmol/L (F)=1 | |
| ***BMD (T value)*** | <–1=0; ≥–1=1 |  |
